# Monocytes and neutrophils promote cardiac fibroblast pro-fibrotic phenotypes through IL-6 and MIF

**DOI:** 10.3389/fcell.2026.1830777

**Published:** 2026-05-12

**Authors:** Zhongxiao Cong, Aleksandar Ivetic, Maddy Parsons

**Affiliations:** 1 Randall Centre for Cell and Molecular Biophysics, King’s College London, Guy’s Campus, London, United Kingdom; 2 School of Cardiovascular and Metabolic Medicine and Sciences, James Black Centre, King’s College London British Heart Foundation Centre of Excellence, London, United Kingdom

**Keywords:** cardiac fibrosis, contraction, cytokine secretion, extracellular matrix, fibroblasts, migration, monocytes, neutrophils

## Abstract

Cardiac fibrosis, characterized by activation of cardiac fibroblasts and accumulation of extracellular matrix (ECM), is associated with most cardiac pathological conditions, leading to adverse cardiac remodeling and accelerating progression of heart failure. Immune cell recruitment is a hallmark of early fibrosis; however, the underlying mechanisms linking early inflammation, fibroblast activation and perpetuation of cardiac fibrosis remain unclear. In this study, cell-derived matrices (CDM) and collagen gels were used to investigate primary human cardiac fibroblast (HCF) pro-fibrotic response, native ECM synthesis and interactions with immune cells. Direct co-culture of HCF with THP-1 and HL-60 cells, or conditioned media from HCF-immune cell co-cultures, resulted in enhanced HCF-induced contraction of collagen gels - a finding recapitulated using primary human monocytes and neutrophils. Pro-fibrotic cytokines, macrophage migration inhibitory factor (MIF) and interleukin-6 (IL-6), were upregulated in HCF-immune co-cultures and were further confirmed as active regulators of HCF contraction using recombinant purified cytokines and function blocking antibodies. Live imaging revealed that THP-1 cells increased HCF migration, which was suppressed by an IL-6 neutralizing antibody. These findings provide insight into the effects of early exposure of HCF to immune cells and shed light on early pro-fibrotic initiation mechanisms and therapeutic targets.

## 1 Introduction

Cardiovascular disease (CVD) is the leading cause of mortality, accounting for approximately 32% of global deaths in 2022 (World Health Organisation, 2025). Cardiac fibrosis, characterized with excessive deposition of extracellular matrix (ECM) proteins, is an integral component of most forms of cardiomyopathy, leading to increased tissue stiffness, disrupted contractile capacity, impaired cardiac function and accelerated progression of heart failure (HF) (Ghazal et al., 2025). However, signatures of pathological progression of cardiac fibrosis are challenging to identify at early stages by non-invasive diagnostic methods (Ravassa et al., 2023). Additionally, once established, fibrotic tissue is considered a biologically stable and irreversible state, making cardiac fibrosis a significant clinical and therapeutic challenge (Bertaud et al., 2023).

Cardiac fibroblasts (CF) are the most abundant cell population in the myocardium, and the major cell type for ECM synthesis and turnover in healthy, homeostatic conditions (Sullivan and Deems Black, 2013). Thus, CF play a crucial role in maintaining cardiac ECM network integrity and geometry. CF exert an essential function in regulating ECM turnover. Under pathological conditions, CF respond to biochemical, mechanical and electrical signals between cellular and acellular components of the heart and transform into active phenotypes to mediate ECM remodeling. Myofibroblasts are considered the fully activated and terminally differentiated phenotype, marked by expression of alpha-smooth muscle actin (α-SMA) (Tai et al., 2021). In comparison to quiescent fibroblasts, myofibroblasts are highly contractile and exhibit increased ECM production, turnover, with enhanced proliferation, migration and cytokine secretion (Younesi et al., 2024). Persistent activation of CF leads to excessive ECM production, resulting in local matrix deposition that progressively alters the myocardial microenvironment (Fan et al., 2012). Dysregulation of ECM also compromises cardiac integrity and cell connectivity, and the resulting mechanical dysregulation leads to diastolic and systolic dysfunction, thereby accelerating the progression of HF (Frangogiannis, 2019).

Growing evidence demonstrates that cardiac remodeling and fibrosis are related to both acute and chronic inflammation. In the heart, resident immune cells and those recruited into the injury area initially remove injurious stimuli and cell debris to promote repair (Silva et al., 2021). Subsequently, immune cells also secrete growth factors or cytokines, promoting CF activation and ECM deposition as well as other tissue resident cells (Rao et al., 2025). Transforming growth factor-beta1 (TGF-β1), is a well characterized mediator of fibrosis, produced by fibroblasts, immune, endothelial and epithelial cells, and promotes CF proliferation, α-SMA expression and ECM deposition (Yousefi et al., 2020). TGF-β1 can also promote cytokine secretion by immune cells to exacerbate fibrosis (Saadat et al., 2021). Monocytes/macrophages and neutrophils dominate the early inflammatory response in cardiac fibrosis, with neutrophils initiating tissue injury responses and monocyte-derived macrophages coordinating the transition from inflammation to fibrosis. In mouse models of myocardial infarction, monocyte-derived macrophages largely replace tissue resident macrophages during the later post-injury stage and correlate with cardiac fibrosis (Bajpai et al., 2019), and inhibition of neutrophil recruitment slows fibrotic progression (Ma, 2021).

Both macrophages and neutrophils respond to damage-associated molecular patterns (DAMP) from damaged CM and rapidly accumulate in the injury site, triggering activation of inflammatory cascades (Lavine et al., 2018). Following injury, recruited neutrophils release proteases for removal of necrotic debris, and cytokines and chemokines to subsequently recruit other immune cells into the injury area (Kolaczkowska and Kubes, 2013). For instance, C-C motif chemokine ligand 2 (CCL2), also referred to as monocyte-chemoattractant protein-1 (MCP-1), contributes to monocyte recruitment (Li and Frangogiannis, 2021), while interleukin-6 (IL-6) promotes CF proliferation and ECM synthesis (Venkatachalam et al., 2009). Excessive accumulation of neutrophils can also lead to increased levels of reactive oxygen species (ROS), contributing to lipid peroxidation, protein oxidation, and DNA damage of CM, followed by cell apoptosis, fibrotic signaling and expansion of infarct area (Ma, 2021). Neutrophil extracellular traps (NET), composed of DNA, histones, and proteins are also released by neutrophils and accumulate in the injury site, promoting CF activation (Zhang et al., 2025) and other pro-inflammatory signaling (Zheng et al., 2025). Resident macrophages in injury area play an essential role in immunosurveillance to eliminate dead cells and debris (Epelman et al., 2014; Li et al., 2021a) and promote neutrophil infiltration and residential macrophage recruitment from uninjured areas (DeLeon-Pennell et al., 2017). Subsequently, increased levels of CCL2 from neutrophils further recruit monocytes from peripheral blood into infarct areas. Macrophages derived from recruited monocytes synthesize pro-fibrotic mediators, such as IL-1, necrosis factor-alpha (TNF-α) and Ang-II (Chen et al., 2024), and promote production of other pro-fibrotic factors from other cardiac cell populations, such as connective tissue growth factor (CTGF) in CF (Lugrin et al., 2023).

Whilst animal models have advanced understanding of CVD (Patten and Hall-Porter, 2009), they do not mimic the early CF-immune cell interactions and complex ECM remodeling in patients (Ding et al., 2020). Moreover, the early interplay between immune cells and CF pro-fibrotic phenotype perpetuation remains incompletely understood. Here we present evidence using complex *in vitro* 3D models with fibroblast-immune co-cultures to overcome limitations of traditional 2D models and link early inflammation to the initiation of cardiac fibrosis. We reveal that both immune cell secreted factors and direct interactions between HCF and immune cells contribute to HCF ECM production and contractile behaviour. We further demonstrate that secreted IL-6 and MIF promote HCF contractility and that macrophages further induce HCF migration to increase fibrotic remodelling. These findings shed light on early induction of pro-fibrotic mechanisms, potential early intervention points and targets for therapeutics.

## 2 Materials and methods

### 2.1 Antibodies

Antibodies used in this study (working final concentrations in brackets for immunofluorescence were as follows: Anti-α-SMA (0.4 μg/mL, Abcam ab5694), anti-Collagen I (0.4 μg/mL, Abcam ab138492), anti-Fibronectin I (10 μg/mL, Sigma-Aldrich F7387), anti-Mouse Alexa Fluor®-488, −568 (4 μg/mL, Invitrogen, A11001and A11004 respectively), anti-Rabbit Alexa Fluor®-488, −568, −647 (4 μg/mL, Invitrogen, A11008, A11011and A22287 respectively). Antibodies used for Western blotting: anti-RhoA (1 μg/mL, Novus 1A11-4G0) and anti-HSC70 (0.2 μg/mL, Santa-Cruz Biotech SC-7298). Anti-IL6 (Bio-techne, AF-206-NA) and anti-MIF (Merck, MABF111) neutralizing antibodies were used at 500 ng/mL.

#### 2.1.1 Cell culture

Primary Human Cardiac Fibroblasts (HCF), isolated from adult heart ventricles were purchased from PromoCell (Passage 1/2, C-12375). Three different lot numbers of cells, each from one donor (2 male, one female, age range 22–59 years) were used for experiments. HCF were grown in Fibroblast Growth Medium 3 (PromoCell) and maintained at 37 °C, 5% CO_2_ in a humidified incubator and passaged upon reaching 90% confluence using TrypLE enzyme (Gibco). THP-1 and HL-60 cells (human monocyte-derived and human promyelocytic cell line respectively) were obtained from ATCC. HL-60 cells were grown in Roswell Park Memorial Institute-1640 (RPMI-1640) growth medium (Sigma-Aldrich), supplemented with 10% FBS, 2 mM ʟ-Glutamine, 100 units/mL penicillin and 0.1 mg/mL streptomycin. THP-1 cells were grown with the same complete RPMI-1640 supplemented with additional 50 μM β-mercaptoethanol (Sigma-Aldrich). Cells were plated at a density of 0.5 × 10^6^/mL and passaged upon reaching approximately 1.5 × 10^6^/mL. Cell counting was performed using Trypan Blue Exclusion Assay on a hemocytometer under brightfield microscope settings. For co-culture, THP-1 cells were activated by incubation with 100 ng/mL human recombinant CCL2 (R&D, 279-MC-050) at a density of 1 × 10^6^/mL in THP-1 culture medium overnight (Ashida et al., 2001). Differentiation of HL-60 to neutrophil-liked HL-60 was performed with 1.3% DMSO (Sigma-Aldrich, D2438) in complete RPMI-1640 at a density of 1 × 10^6^/mL for 4 days (Rahman et al., 2021).

#### 2.1.2 Primary immune cell isolation

Peripheral blood samples were obtained from healthy donors under King’s College London internal ethical approval REMAS-24/25-20917 (Research Ethics Management Application System). Peripheral blood cells were separated using density gradient centrifugation (Cui et al., 2021), 25 mL of whole blood was layered on 15 mL of Histopaque (Sigma-Aldrich) and then centrifuged at 300 × g for 30 min with the lowest acceleration and deceleration. After removing the top plasma layer, the ‘buffy coat’ layer, containing human peripheral blood mononuclear cells (PBMC), was collected and washed twice with 50 mL of Hank’s balanced salt solution (HBSS, Sigma-Aldrich), followed by centrifugation at 300 × g for 5 min for monocyte collection. The histopaque layer beneath ‘buffy coat’ was discarded, and the red blood cell (RBC) layer was diluted with HBSS at ratio 1:1 in a new 50 mL falcon tube. The RBC/HBSS mixture was mixed with an equal volume of filter-sterile 2% Dextran (w/v) and incubated for 30 min at room temperature for further neutrophil isolation. Buffer Solution was prepared by diluting 0.5% BSA stock solution in AutoMACS Rinsing solution at a ratio of 1:20. CD14^++^CD16^−^ monocytes were isolated with the Classical Monocyte Isolation Kit (Miltenyi Biotec). PBMC pellets were resuspended with 300 μL of pre-cold Buffer Solution and mixed with 100 μL of Classical Monocyte Biotin-Antibody Cocktail, 100 μL of CD61 Microbeads and 50 μL of Thrombocyte Removal Reagent and then incubated for 5 min at room temperature. Cells were then incubated with 300 μL of Buffer Solution and 200 μL of Anti-Biotin Microbeads for another 5 min at room temperature. The LS column was placed in the magnetic field of the MACS Separator and rinsed with 3 mL of Buffer Solution. The PBMC suspension was then washed with 3 mL of Buffer Solution. The flow though containing CD14^+^CD16^−^ monocytes was collected and centrifuged at 300 × g for 5 min at 4 °C. Monocytes were then washed 3 times with HBSS followed by centrifuging 300 × g for 5 min at 4 °C. The cell pellet was then resuspended with 1 mL of HBSS and placed on ice for further use. For neutrophil isolation, the top opaque layer of RBC mixture was retained and mixed with an equal volume of HBSS. The cell/HBSS mixture was then diluted with equal amount of amount of 2% Dextran (w/v in HBSS) and left at room temperature for 30 min. After centrifugation at 300 × g for 5 min with lower acceleration and deceleration, the RBC pellet was then resuspended with 3 mL of sterile water and incubated for 30 s to break down the remaining red blood cells. The break-down was ended by adding 47 mL of HBSS and the neutrophils were collected by centrifugation at 300 × g for 5 min with lower acceleration and deceleration. The neutrophil pellet was resuspended with 5 mL of HBSS and stored on ice for further use.

#### 2.1.3 Cell derived matrices

Methods were adapted from (Kaukonen et al., 2017). Briefly 13 mm glass coverslips were sterilized by soaking in 70% industrial methylated spirits (IMS) and coated with 800 μL of gelatin (0.2% w/v in PBS) (Sigma-Aldrich, 04,055) 1 h at 37 °C or overnight at 4 °C. After washing 3 times with PBS, gelatine was then cross-linked with filter-sterilized glutaraldehyde (1% v/v in PBS) (Bio Basic) by incubating 30 min at room temperature. PBS washing was performed 3 times, followed by incubation with 500 μL of 1 M glycine (Sigma-Aldrich) 20 min at room temperature to quench remaining glutaraldehyde. Before using, coverslips were incubated with complete DMEM for 1 h at 37 °C. HCF were seeded with fibroblast growth medium at a density of 5 × 10^4^ cells/well and maintained 1–2 days until cells formed a confluent monolayer (∼2 × 10^5^/well). Culture medium was then replaced with complete RPMI-1640 (2.5% FBS, supplemented with 50 μg/mL filtered ascorbic acid) (Sigma-Aldrich, A4544) for initial treatment or immune cell co-culture and then changed every other day with complete DMEM (2.5% FBS, supplemented with 50 μg/mL filtered ascorbic acid). HCF were treated with 10 ng/mL TGF-β1 or co-cultured with THP-1/HL-60 at 1:5 ratio (immune cell:HCF, ∼4 × 10^4^ cells/well) for the first 48 h. TGF-β1 treatment or immune cells were removed at the first medium change, verified visually under a brightfield microscope. After removing treatments, cells were grown for a further 10 days before analysis.

#### 2.1.4 Fibroblast-immune cell co-culture conditioned media

HCF were seeded in 6-well plates at a density of 1.5 × 10^6^ cells/well using fibroblast growth medium and maintained until cells had reached to approximately 90% confluency (∼1 × 10^6^ cells/well). THP-1 or HL-60 cells were then added on top of HCF monolayers at 1:5 ratio (immune cell:HCF, ∼2 × 10^5^/well), respectively. Medium was replaced with 3 mL of complete RPMI-1640 (2.5% FBS) or OptiMEM (Gibco, 11058-021) depending on onward experiments. Conditioned media were collected after 48 h of incubation with immune cells and were filtered with a 0.45 μM filter for further use.

#### 2.1.5 Collagen gel contraction and imaging

For collagen gel contraction assays, method was adapted from a published protocol (Bell et al., 1979). Rat tail collagen type I stock (Sigma-Aldrich, 354,249) at concentration of 9–11 mg/mL was supplemented with 3% 1 M NaOH and then mixed with cell suspension to make the final cell density at 2.5 × 10^4^/mL and final collagen concentration of 0.85 mg/mL. 410 μL of cell-collagen mixture (+/-immune cells at ratio 1:1) were seeded into each well of 48-well plates. The plate was then incubated at 37 °C for 15 min to polymerize the gel. After gently releasing the gels from edges with a pipette tip, equal volumes of fresh complete RPMI-1640 (2.5% FBS) or conditioned media in complete RPMI-1640 (2.5% FBS) was then added into each well. Additional treatments (purified cytokines or neutralizing antibodies) were supplemented into fresh complete RPMI-1640 (2.5% FBS) as needed. Cells were then placed back into incubator and cultured at 37 °C for 5 days. Images were taken with BioRad ChemiDoc Imaging system every 24 h. The surface area of each gel was manually selected and measured. For co-culture experiments in 3D gels and live imaging, HCF suspension in complete RPMI-1640 (2.5% FBS) (+/-immune cells at ratio 1:1) was mixed with rat tail collagen type I stock, supplemented with 4 mM NaOH, 10 μg/mL fibronectin, 20 mM HEPES, 0.3% NaHCO3, to make a final cell density at 2 × 10^4^ cells/mL and a collagen concentration of 2 mg/mL. 100 μL of cell-collagen mixture was then seeded into each well of 96-well imaging plates. After incubating at 37 °C for 10 min, the plates were flipped and cultured for another 10 min to prevent cells settling at the bottom until the gels were fully polymerised. For lack of external CO_2_ during microscopy imaging, 300 μL of complete RPMI-1640 (2.5% FBS), supplemented with 25 mM HEPES (Itagaki and Kimura, 1974) was added. Images were taken every 45 min for in total 24 h.

### 2.2 Cytokine array

Quantification of cytokine and chemokines was undertaken on freshly collected conditioned media using the Proteome Profiler Human Cytokine Array Kit (Bio-techne, ARY005B) according to the manufacturer’s guideline. Briefly, 1 mL of each sample was mixed with 500 μL Array Block Buffer and 15 μL of reconstituted Human Cytokine Array Detection Antibody Cocktail and incubated at room temperature for 1 h. Membranes were placed on the 4-well multi-dish and incubated with 2 mL of Array Block Buffer for 1 h at room temperature on a rocking platform shaker, followed by an overnight incubation with sample/antibody mixtures at 4 °C on a rocking platform. After washing 3 times for 10 min, the membrane was incubated with 2 mL of diluted Streptavidin-HRP for 30 min followed by washing 3 times. The membrane was incubated with 1 mL of freshly made Chemi Reagent Mix for 1 min. Signals were then visualized by exposing the membrane to UV light for 1–10 min with ChemiDoc imaging system. To identify positive signals on array images, the transparency overlay template provided was placed and aligned with the pairs of reference spots in the three corners. The pixel density per spot was analyzed using BioRad Image Lab 6.0 and the adjusted volume for each spot was acquired; a background region from a clear area of the membrane wa also analyzed and subtracted from the values for each cytokine spot. Cytokine signals were subsequently normalized against positive reference spots and pixel densities between control and untreated samples were calculated to determine fold change.

### 2.3 Enzyme-linked immunosorbent assay (ELISA)

ELISAs were performed using Human IL-6 and MIF ELISA Kits (Ray Biotech, ELH-IL6-1 and ELH-MIF-1 respectively) according to manufacturer’s guidelines. Standard samples (IL-6 from 0 to 1 ng/mL, MIF from 0 to 6 ng/mL) were prepared by gradient dilution with standard protein provided by the kit. Appropriate numbers of wells were placed on the detachable 8-Strip ELISA plate frame and incubated with 100 μL of each standard and sample for 2.5 h at room temperature with gentle shaking. The wells were washed 4 times with 300 μL of 1X wash solution and emptied properly by flicking vigorously, followed by a 1-h incubation with 100 μL of cytokine detected antibody (diluted with assay dilution buffer as required) at room temperature with gentle shaking. The wells were washed as described above and incubated with 100 μL of 1X HRP-Streptavidin solution for 45 min at room temperature with gentle shaking. Each well was incubated with 100 μL of TMB One-Step Substrate Solution Reagent after washing as before for another 30 min at room temperature in the dark before 50 μL of Stop Solution was added, and the plate was immediately read at 450 nm on the POLARstar Omega Microplate Reader.

#### 2.3.1 Immunostaining

To detect and visualize specific proteins, immunofluorescence (IF) staining was performed on fixed CDM samples. CDM on coverslips were fixed with 500 μL of 4% PFA in PBS (pH 7.4) for 15 min at room temperature and washed with PBS 3 times, followed by incubation with 500 μL of 0.2% Triton-X100 in PBS (v/v) for 10 min at room temperature to permeabilize cell membrane. After 3-times PBS wash, samples were then blocked with 500 μL of 3% BSA in PBS (w/v) for 1 h at room temperature. Primary antibody incubation was performed after blocking by incubating with appropriate antibodies diluted with blocking buffer overnight at 4 °C. After washing with PBS for 3 times, coverslips were then incubated in the dark with appropriate secondary fluorescent conjugated antibodies (diluted with blocking buffer), supplemented with Hoechst and Phalloidin, overnight at 4 °C. This was followed by washing with PBS 3 times and MilliQ water once, and coverslips were then mounted onto labelled glass slides with Fluorsave™ mounting reagent and dried in the dark.

To monitor interactions between live HCF and immune cells in collagen gels, cells were labelled with 4 μM CellTracker Green CMFDA (ThermoScientific, C7025) and 4 μM CellTracker Orange CMTMR (ThermoScientific, C34551) in FBS free DMEM for HCF or RPMI-1640 for immune cells separately for 30 min at 37 °C before seeding into collagen gels. SPY650 DNA (SPIROCHROME, SC501) was diluted with complete RPMI-1640 and added onto collagen gels after polymerization for at least 1 h before imaging.

#### 2.3.2 Microscopy

Imaging of fixed CDM and live cells seeded into collagen gels was performed using a Nikon AIR inverted confocal microscope (Nikon Instruments United Kingdom) equipped with a 37 °C environmental chamber. Excitation wavelengths of 405 nm (diode laser), 488 nm (argon laser), 561 nm (diode laser) and 640 nm (diode laser) were used. Images for CDM samples were taken using a ×40 Plan Fluor oil immersion objective while images of cells in collagen gels were taken using a ×40 S Plan Fluor ELWD objective. 0.225 μm z-stacks were acquired to capture the CDM volume. To evaluate the migration of HCF and immune cells over time during co-culture, images from five different fields of view per gel were taken consecutively every 45 min for 24 h. Images were acquired with NIS-Elements imaging software and saved in. nd2 format for further analysis.

#### 2.3.3 Image analysis

To measure the intensity of collagen I and fibronectin, images were imported into FIJI (v2.16.0) and separated into single channels where relevant. Each image was evenly divided into 16 equal regions to allow for sampling of the spatial heterogeneity of the CDM model. The mean intensity of each ROI was measured using the ‘measure’ function in FIJI (v2.16.0), followed by export of data into Prism (v10.5.0) for processing.

For cell migration analysis in collagen gels, images were taken every 45 min for 24 h. All images were processed in FIJI (v2.16.0) using the manual tracking function to track nuclei of single cells until cells became stationary or moved beyond the imaging area. Results with coordinates for directionality, distance and velocity were provided and imported into Chemotaxis and Migration Tool, a FIJI plugin developed by Ibidi®, to obtain the migration rose-plots, mean velocity (μm/min) and directionality of single cells. Data was exported into prism for statistical analysis.

## 3 Results

### 3.1 Early drivers of fibrosis are triggered by cardiac fibroblast co-culture with monocytes and neutrophils

**FIGURE 1:**
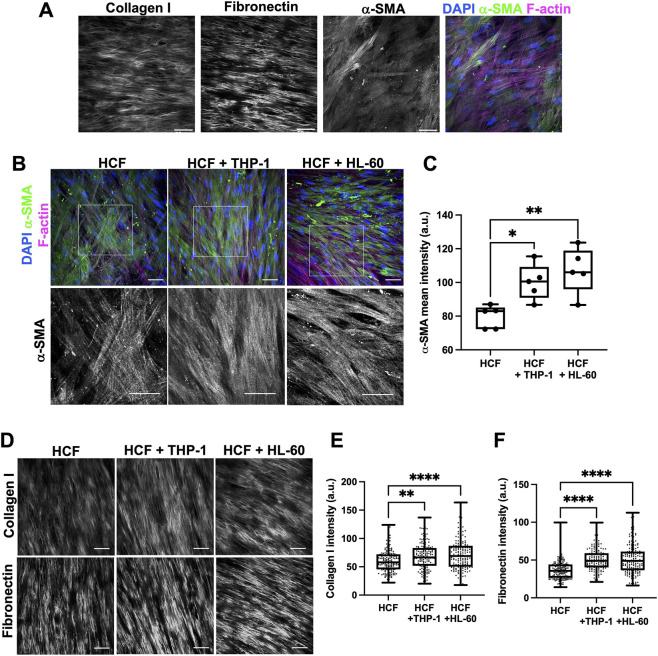
Acute exposure to both THP-1 and HL-60 cells induces increases HCF collagen I and fibronectin deposition in cell-derived matrices. **(A)** Example single channel images of collagen I, fibronectin and α-SMA (all shown in white), and merged nuclei (blue; DAPI), α-SMA (green; Alexa-488) and F-actin (magenta; Alexa-568) of HCF in CDM after 12 days culture. **(B)** Representative merged images of nuclei (blue; DAPI), α-SMA (green; Alexa-488) and F-actin (magenta; Alexa-568) of HCF in CDM after 12 days, cultured for first 2 days with noted immune cells. α-SMA channel is shown below each in white. Scale bars represent 50 μm. **(C)** Quantification of mean intensity from images as in **(B)**. Data shown is from one experiment, representative of three independent experiments (5 fields of view per coverslip, per condition). **(D)** Representative images of collagen I and fibronectin in CDM with or without immune cell (THP-1 or HL-60) co-culture for the first 2 days; quantification of local mean intensity for **(E)** collagen I and **(F)** fibronectin. Data shown is from one experiment, representative of three independent experiments. n = 160 regions divided from 10 fields of view from one coverslip analyzed per condition. Statistical analysis in C, E, F performed by one-way ANOVA, followed by Dunnett’s multiple comparisons test. *p < 0.05 **p < 0.005, ****p < 0.0001.

Cell-derived matrices (CDM) are established models to study native ECM deposition and fibroblast behavior in a more physiologically relevant environment (Franco-Barraza et al., 2016). Primary human cardiac fibroblasts (HCF) produced abundant collagen and fibronectin and exhibited pro-fibrotic α-SMA expression in CDM (Figure 1A). TGF-β1 alone induced α-SMA expression, but only when used with low (2.5%) serum, as high (10%) serum masked any effects of this growth factor (Supplementary Figure S1A,B). TGF-β1 in 2.5% serum cultures led to collagen and fibronectin deposition in CDM after 12 days of culture (Supplementary Figure S1C,D). To test roles for early inflammation, we used THP-1 and HL-60 cells as models for monocyte/macrophages and neutrophils respectively. Co-culture of these cells with HCF for the first 2 days of CDM production resulted in increased HCF α-SMA levels (Figures 1B,C) and higher collagen and fibronectin deposition (Figures 1D–F). Co-culture of HCF and THP-1 cells induced higher proliferation in HCF in 2D cultures with no change seen with TGF-β1 treatment or HL-60 cell co-culture (Supplementary Figure S2A,B). Further analysis of images revealed THP1 cells induced elongation of HCF, whereas HL-60 promoted more rounded phenotypes (Supplementary Figure S2C). These data demonstrate that short-term exposure of HCF to monocyte and neutrophil models promotes HCF contractile phenotypes, ECM deposition and proliferation, all hallmarks of early fibrosis.

### 3.2 Cardiac fibroblast collagen gel contraction is enhanced by immune cells

**FIGURE 2:**
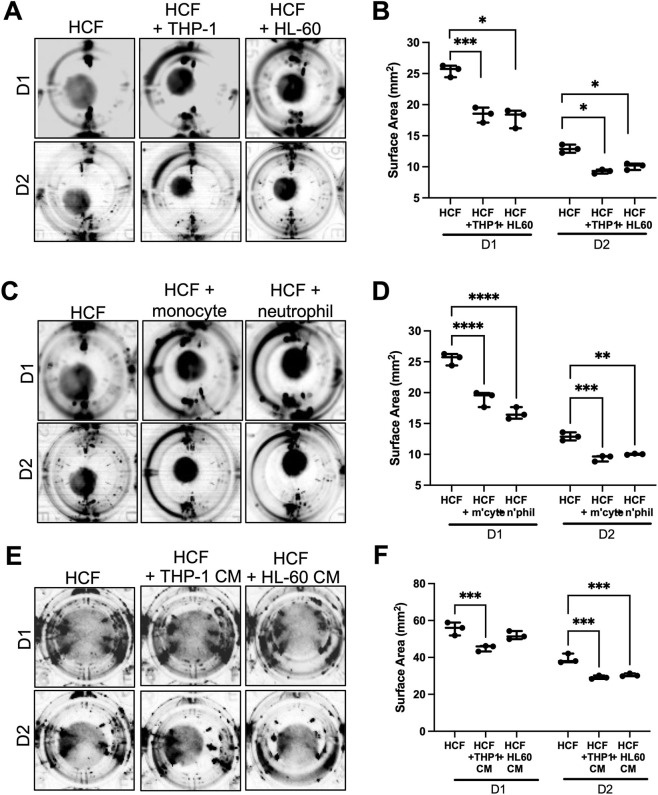
Direct and indirect co-culture with monocytes or neutrophils enhances HCF collagen gel contraction. Representative images of collagen gels over 2 days containing HCF alone or **(A)** co-cultured with immune cell lines THP-1 and HL-60, **(C)** co-cultured with human primary monocytes and neutrophils and **(E)** cultured with conditioned media (CM) from HCF-THP-1 or HCF-HL-60 cell co-cultures. **(B, D, F)** Quantification of collagen gel surface area over time for **(A, C, E)** respectively. Data shown is from one experiment (n = 3 gels per condition), representative of three independent experiments; statistical analysis performed by two-way ANOVA, followed by Bonferroni’s multiple comparisons test. *p < 0.05, **p < 0.01, ***p < 0.001.

To further investigate functional consequences of immune cells on HCF contractile behavior, cells were co-cultured in collagen I gels and contraction analyzed over 2 days. HCF alone resulted in a baseline of contraction at day 1, with greater contraction seen at day 2 (Figures 2A,B). Addition of both THP-1 and HL-60 cells induced greater contraction at day1 compared to HCF alone, and this persisted at day 2 post-seeding (Figures 2A,B). The same findings were seen using primary monocytes and neutrophils isolated from fresh human peripheral blood (Figures 2C,D). To test whether direct cell-cell interactions were required, HCF were incubated with conditioned media from HCF co-cultures with either THP-1 cells or HL-60 cells. Both conditioned media types induced significantly greater collagen gel contraction by HCF vs. HCF in normal growth media alone (Figures 2E,F).

Both immune cell types induced α-SMA expression in HCF (Figure 1) which is known to promote HCF contractility. As RhoA is a mediator of HCF contractility (Santos et al., 2019), RhoA GTPase activation, total RhoA expression and activation were measured in HCF lysates with or without immune cell co-culture. RhoA levels did not change between HCF alone or treated with TGF-β1 or immune cells (Supplementary Figure S3A,B). Similarly, RhoA activation measured by G-LISA was also unchanged across conditions (Supplementary Figure S3C). Treatment of HCF with conditioned media from HCF-immune cell co-cultures also did not change RhoA expression (Supplementary Figure S3D,E) or activation (Supplementary Figure S3F). Activation of Rho Kinase (ROCK) - a RhoA effector kinase - was required for HCF induced collagen gel contraction, both alone and with immune cell co-culture, although notably contraction remained elevated in THP-1 and HL-60 cell co-cultures in the presence of ROCK inhibitor compared to HCF alone (Supplementary Figure S3G,H). These data collectively demonstrate that monocytes and neutrophils promote HCF-induced collagen gel contraction which is in part mediated through ROCK activity.

### 3.3 Monocytes and neutrophils promote cardiac fibroblast secretion of IL-6 and MIF

**FIGURE 3:**
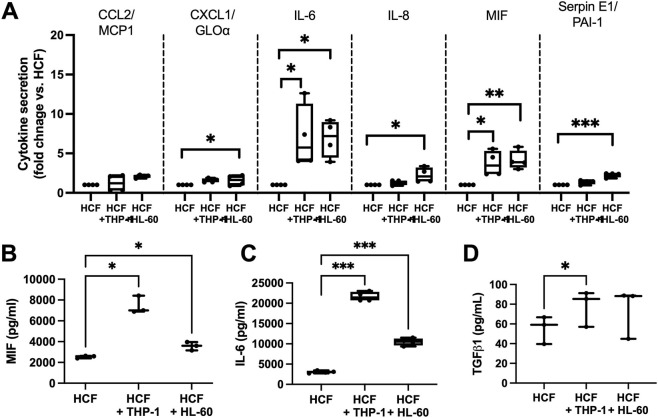
Co-culture with immune cells induces HCF IL-6 and MIF release. **(A)** Quantification of cytokines detected from cytokine array. Statistical analysis performed by one-way ANOVA, followed by Dunnett’s multiple comparisons test. n = 4 experiments shown. *p < 0.05, **p < 0.01, ***p < 0.001. **(B–D)** Quantification of ELISAs for MIF, IL-6 and TGF-β1 respectively in conditioned media from HCF with or without immune cell co-cultures for 48 h. Statistical analysis performed by one-way ANOVA, followed by Dunnett’s multiple comparisons test. n = 3 independent experiments shown for B-D. *p < 0.05, ***p < 0.001.

As shown in Figure 2F, conditioned media promoted HCF-induced collagen gel contraction, indicating that secreted factors from HCF-immune cell co-cultures may promote fibroblast contractile behavior. Analysis of conditioned media from HCF alone or co-cultured with THP-1 or HL-60 cells using a cytokine array revealed release of seven key pro-inflammatory cytokines and chemokines from cells (Supplementary Figure S4A,B). Of these seven detected factors, interleukin-6 (IL-6) and macrophage migration inhibitory factor (MIF) were both significantly elevated in conditioned media from HCF-THP-1 and HCF-HL-60 cultures vs. HCF alone (Figure 3A). CXCL1, IL-8 and Serpin E1 were also increased in media from HCF-HL-60 cultures only vs. HCF alone (Figure 3A). Elevated release of MIF and IL-6 in HCF-immune co-cultures was confirmed using quantitative ELISA (Figure 3B,C respectively). Additional analysis of secreted TGF-β1 revealed an increase in secretion of this growth factor in HCF-THP-1 co-cultures vs. HCF alone, with no significant change seen in HL-60 co-culture media (Figure 3D). These findings collectively demonstrate that HCF exposure to monocytes or neutrophils consistently promote IL-6 and MIF release, both of which are implicated in pro-fibrotic phenotypes.

### 3.4 IL-6 and MIF promote cardiac fibroblast collagen contraction and migration

**FIGURE 4:**
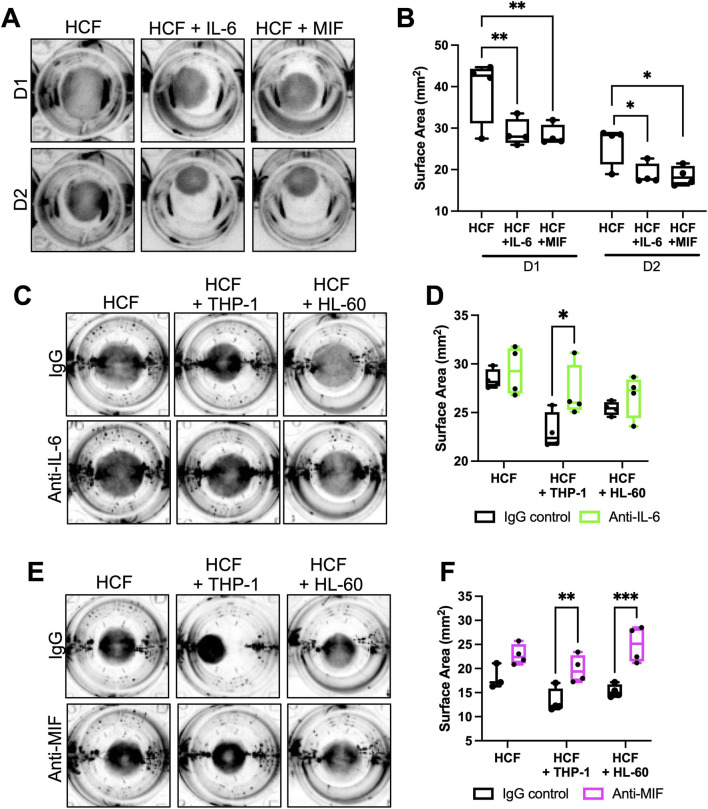
IL-6 and MIF release promote HCF-induced collagen gel contraction. **(A)** Representative images of collagen gels containing HCF treated with recombinant IL-6 (100 ng/mL) or MIF (20 ng/mL) over 2 days **(B)** Quantification of collagen gel surface area from images as in **(A)**. **(C,E)** Representative images of collagen gels containing HCF co-cultured with immune cells treated with IgG control antibody or **(C)** anti-IL6 or **(E)** anti-MIF at day 2. **(D,F)** Quantification of collagen gel surface area from images as in **(C,E)** respectively. Data shown in B, D and F is from one experiment (4 samples per condition), representative of three independent experiments. Statistical analysis performed by **(B)** one-way ANOVA, followed by Dunnett’s multiple comparisons test and **(D,F)**, two-way ANOVA, followed by Bonferroni’s multiple comparisons test. *p < 0.05, **p < 0.01, ***p < 0.001.

**FIGURE 5:**
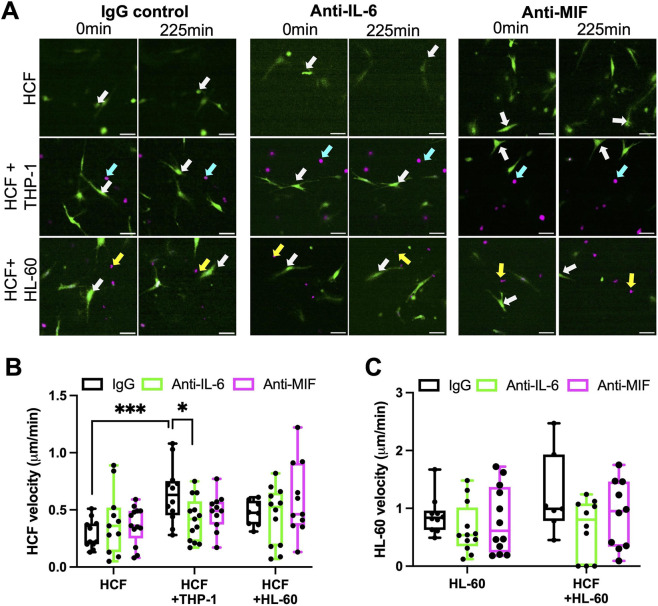
IL-6 promotes THP-1-induced HCF migration in 3D collagen gels. **(A)** Representative images of stills from confocal live cell time lapse movies of HCF alone (green, Alexa-488) or co-cultured with THP-1 (magenta, cyan arrows) or HL-60 (magenta, yellow arrows) cells embedded in 3D collagen gels. Cultures were treated with IgG or IL-6/MIF neutralizing antibodies (0.5 μg/mL) from start of experiment. Scale bar represents 50 μm. **(B,C)** Quantification of velocity over imaging period with or without IL-6 or MIF neutralizing antibody **(B)** HCF alone and in presence of immune cells and of **(C)** HL-60 alone and in presence of HCF. Data shown from one experiment (n = 7–13 movies tracked per condition with >3 cells per field of view), representative of three independent experiments; statistical analysis performed by two-way ANOVA, followed by Tukey’s multiple comparisons test. *p < 0.05, ***p < 0.001.

To determine whether IL-6 and MIF were sufficient to induce pro-fibrotic contractile phenotypes, HCF in collagen gels were treated with recombinant IL-6 (100 ng/mL) or MIF (20 ng/mL) and contraction measured over 2 days. Both IL-6 and MIF promoted significantly higher contraction of collagen gels by HCF compared to untreated HCF at both 1 and 2 days post-seeding of cells (Figures 4A,B) indicating early triggering of pro-fibrotic behavior. Moreover, neutralizing antibodies to both IL-6 (Figures 4C,D) and MIF (Figures 4E,F) both significantly reduced collagen gel contraction in HCF-THP-1 co-cultures over the 2-day measurement period, and MIF neutralization also suppressed contraction in HCF-HL-60 cell cultures. To further explore the relationship between HCF and immune cells in 3D collagen gels, cells were cultured alone or with HCF in combination with THP-1 or HL-60 cells and behavior analyzed using time-lapse confocal microscopy (Figure 5A). Tracking of cell types cultured alone in 3D matrices revealed that HL-60 cells exhibited the fastest migration speeds, with HCF exhibiting slower migration than HL-60 and THP-1 cells migrated very little during the imaging period (Supplementary Figure S5A,B). Co-culture with THP-1 cells induced significantly faster HCF migration speed compared to HCF alone whereas HL-60 cells did not induce a significant increase in HCF velocity (Supplementary Figure S5C,D). Co-culture with HCF did not change migration speed of either HL-60 cells (Supplementary Figure S5E) or THP-1 cells (Supplementary Figure S5F).

To explore whether secreted IL-6 or MIF that induced HCF contractile phenotypes also contributed to migratory phenotypes, cells were incubated with IL-6 or MIF neutralizing antibodies and migration tracked over time (Figure 5A). Anti-IL-6 treatment significantly reduced HCF migration speed when co-cultured with THP-1 cells compared to IgG control treated cultures (Figure 5B). No significant changes were seen in HCF migration with anti-IL-6 when co-cultured with HL-60 cells, or in either co-culture with anti-MIF (Figure 5B). Moreover, IL-6 and MIF inhibition had no effect on HL-60 cell migration (Figure 5C) indicating specific effects on HCF. These data demonstrate that exposure of HCF to monocytes induces rapid increases in HCF migration speed and that secreted IL-6 contributes to this phenotypic change. These findings, coupled with induction of contractile behavior over 2 days and ECM deposition over 12 days indicate a temporal cascade following early exposure to IL-6 and MIF that trigger pro-fibrotic phenotypes in HCF.

## 4 Discussion

In this study, we demonstrate that secreted IL-6 and MIF from neutrophils and macrophages promote contractile and migratory phenotypes in primary HCF, concurrent with native ECM deposition - all of which are hallmarks of early fibrosis. 3D multicellular culture models, including those used in this study, capture features that are closer to the complex *in vivo* conditions and thus potentially more translatable into clinical applications (Ravi et al., 2015). Increased MIF levels have been found in fibrotic heart tissues from patients (Xue et al., 2018) and IL-6 levels are increased in plasma of patients with chronic heart failure (Birner et al., 2007). As a single ECM component cannot fully mimic features of the *in vivo* cellular environment, CDM models that are secreted and organized by fibroblasts can partially address this problem (Castaldo et al., 2013). Results here demonstrate that acute immune cell exposure induced α-SMA expression and Collagen I/fibronectin deposition in a CDM model. Short-term exposure to immune cells may not recapitulate *in vivo* settings where immune cells can persist, dynamically shift in phenotype, and continuously influence fibroblast behavior throughout the course of injury and repair. Moreover, the model does not account for the contributions of other cell types that are present in cardiac tissue, or any potential contribution from sex-specific factors that may influence phenotypic responses. Most studies investigating sex-specific effects of cardiac fibroblast-immune cell crosstalk have been conducted in murine models or cells, further limiting our understanding of this as a potential confounder *in vitro*. Development of more complex, longer term models including those derived from human inducible pluripotent stem cells (hiPSCs) (Pachter et al., 2025; Stephanie and Vander Roest, 2025) that also consider sex-dependent effects may help to provide greater insight into crosstalk with other cell types in early fibrosis. Moreover, emerging studies detailing the precise ECM composition of fibrotic CVD (Lunde et al., 2024; Buck et al., 2025) are providing greater understanding of dominant features needed to model disease progression *in vitro* - noting however that tissue samples from patients at early disease stages remain a rare resource.

ECM contraction mediated by contractile capacities of activated fibroblasts is critical to accelerate wound closure with higher tension and reduce scar area after injury (Deb and Ubil, 2014). Collagen I is the most abundant ECM protein in human heart tissue, and data shown here demonstrated both direct and indirect co-cultures with either THP-1 or HL-60 and primary neutrophils or monocytes enhanced HCF-induced collagen I matrix contraction. We further assessed whether RhoA–a well characterized small GTPase involved in actomyosin contractility (Santos et al., 2019) – and its downstream effector kinase ROCK may act downstream of immune cell exposure to elicit the pro-contractile effects we observed. Mechanistically, ROCK inhibition reduced HCF contraction but did not prevent the pro-contractile effects of immune cells; no changes were seen in RhoA activation, suggesting that RhoA/ROCK partly regulates HCF contractility. This data agrees with previous work showing TGFβ induces RhoA-dependent α-SMA promoter activation with only partial contributions from ROCK (Masszi et al., 2003) and indicates that additional as yet unexplored mechanisms can increase α-SMA in HCF downstream of immune cell exposure. Our data demonstrates that IL-6 secreted from immune cells also contributes to HCF contraction. Previous studies using rat cardiac fibrosis models reported a role for IL-6 in mediating myofibroblast transitions and collagen production (Meléndez et al., 2010), and knockout of IL-6 in mice prevented cardiac fibrosis formation in a systemic hypertension model (González et al., 2015). IL-6 also increased α-SMA expression in mouse dermal fibroblasts (Gallucci et al., 2006) and ECM expression in human skin fibroblasts (Ghazizadeh et al., 2007). In the former study, ERK1/2 was identified as a downstream mediator of IL-6 induced α-SMA expression. Future studies to dissect the potential co-operativity between ROCK and ERK1/2 in physiological models of immune-cell induced fibroblast contractility may help to determine relative temporal contributions from these pathways to early fibrosis onset. As our study also did not assess IL-6 and MIF contributions to early disease features *in vivo*, this may be an additional interesting area to explore in future. Notably however, rodent models of cardiac fibrosis, such as transverse aortic constriction or coronary artery ligation, typically induce rapid and acute fibrosis, compared to the more subtle transitions in early chronic human disease. The development of new *in vivo* models that reflect the slower onset and lower grade inflammation predicted in human disease settings will be important to provide routes to test early mediators and improve confidence in use for therapeutic testing.

Cell migration towards areas of injury is another feature of reparative and fibrotic phases (Jackson and Artlett, 2025). Data presented here demonstrates that the presence of THP-1 cells - but not HL-60 cells - contributed to enhanced migration of HCF in 3D collagen. Similar findings have been seen using fibroblast-like synoviocytes that exhibited enhanced migration in the presence of THP-1 cells (Ou et al., 2011). Moreover, IL-6 has been shown to increase migration of fibroblasts on 2D surfaces (Shen et al., 2017), and mesenchymal stem cells in 3D collagen (Casson et al., 2018). However, given that both immune cell types secrete IL-6 and MIF, this suggests that additional factors released by THP-1 cells/macrophages may contribute to migratory phenotypes in HCF. Several additional cytokines beyond MIF and IL-6 were detected in co-cultures with specific immune cell types. Levels of IL-8 and SerpineE (plasminogen activator inhibitor-1, PAI-1) were higher in HL-60 co-cultures compared with other groups. Increased expression of IL-8 has been shown in various fibrotic diseases, such as liver, lung and cardiac fibrosis (Zimmermann et al., 2011; Papiris et al., 2018; Zhang et al., 2019). Studies using human lung fibroblasts indicated that IL-8 promoted cell proliferation and migration (Li et al., 2021b). PAI-1 is a serine protease inhibitor and a mediator of fibrinolysis (Shaikh et al., 2024), which could protect ECM from degradation, the sustained activities of which would lead to ECM deposition (Ghosh and Vaughan, 2012). Whether PAI-1 could directly act on HCF *via* ECM remodeling remains unclear. Future experiments investigating IL-8 and PAI-1 roles in contractile and migration behavior may help to dissect temporal contributions from these factors alongside MIF and IL-6 in HCF pro-fibrotic behavior. Moreover, our study did not assess potential signaling pathways triggered in HCF through immune-cell specific secreted factors. In addition to ROCK and ERK, mentioned above, canonical IL-6 receptor activation drives formation of dimers with gp130, activating downstream JAK/STAT and PI3K/Ark, potentially promoting fibrosis in rodent models (Meléndez et al., 2010). Assessing the temporal activation of specific molecular pathways in HCF following 3D co-culture with relevant immune cells in future will enable a broader understanding of early signaling events leading to progressive, chronic fibrosis.

Both THP-1 and HL-60 cells promote HCF pro-fibrotic phenotypes; however, THP-1 cells play a dominant role in controlling HCF migration and contraction in our models. Notably however, neutrophils are far more abundant than monocytes in humans, comprising 55%–70% of circulating leukocytes compared with only 2%–8% for monocytes (Rosales, 2018). The quantities of those two types of cells that attracted to sites of injury have not been fully resolved. In our co-culture experiments these two immune cell types were introduced in equal numbers, which may not fully reflect their physiological ratios *in vivo*. Thus, establishing more physiologically relevant ratios of HCF:immune cells in future, and potentially based on emerging spatial data from cardiac disease tissue, may reveal the balance of signals from each compartment in controlling HCF activation.

In summary, this project provides new insight into the pro-fibrotic effects of initial immune-fibroblast interactions within 3D culture systems, contributing to fibroblast-myofibroblast transition and ECM deposition. These processes are coupled with marked alterations in fibroblast morphology, contractility and motility, highlighting the functional changes in fibrotic progression. IL-6 and MIF have been identified as key regulators of early monocyte- and neutrophil-regulated inflammatory responses. These findings provide potential targets for early therapeutic intervention to attenuate cardiac fibrosis formation.

## Data Availability

The original contributions presented in the study are included in the article/Supplementary Material, further inquiries can be directed to the corresponding author.
