# Supplementary material for: Monocytes and neutrophils promote cardiac fibroblast pro-fibrotic phenotypes through IL-6 and MIF
Source: Front Cell Dev Biol. 2026 May 12;14:1830777. doi: 10.3389/fcell.2026.1830777 (PMC13201410; doi:10.3389/fcell.2026.1830777)
Supplement: Supplementary file 1 [file DataSheet1.pdf]

Supplementary Figure 1

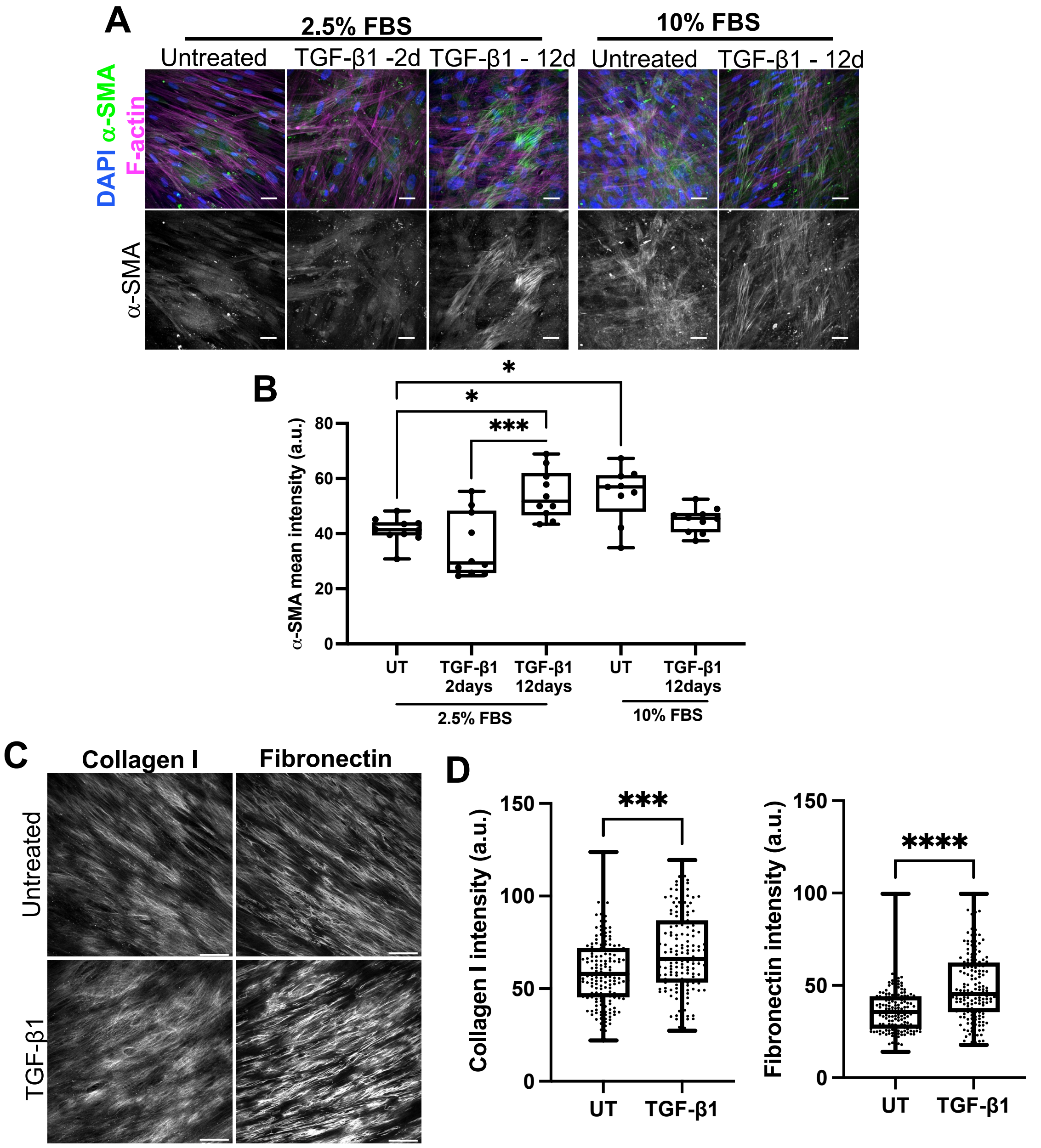

Supplementary Figure 1: TGF-β1 promotes HCF pro-fibrotic phenotypes in cell derived matrices

(A) Representative images of α-SMA staining in HCF in CDM (12-days total culture, +/- TGF-β1 treatment for noted times) with 2.5% or 10% FBS. α-SMA-Alexa488 shown in green, Phalloidin-Alexa568 shown in magenta, DAPI shown in blue. **(B)** Quantification of mean intensity of α-SMA from images as in (A). **(C)** Representative images of collagen I and fibronectin in CDM with 2 days of TGF-β1 treatment. **(D)** Quantification of mean intensity of collagen I and fibronectin from images as in (C). Scale bars represent 50μm. Data shown is from one experiment, representative of 3 independent experiments. n=10 filed of view per condition in (B) and n=160 regions divided from 10 fields of view analysed per condition in (D). Statistical analysis performed by (B) one-way ANOVA, followed by Tukey's multiple comparisons test and (D) unpaired t-test with Dunns . *$p<0.05$, ***$p<0.001$, ****$p<0.0001$.

Supplementary Figure 2

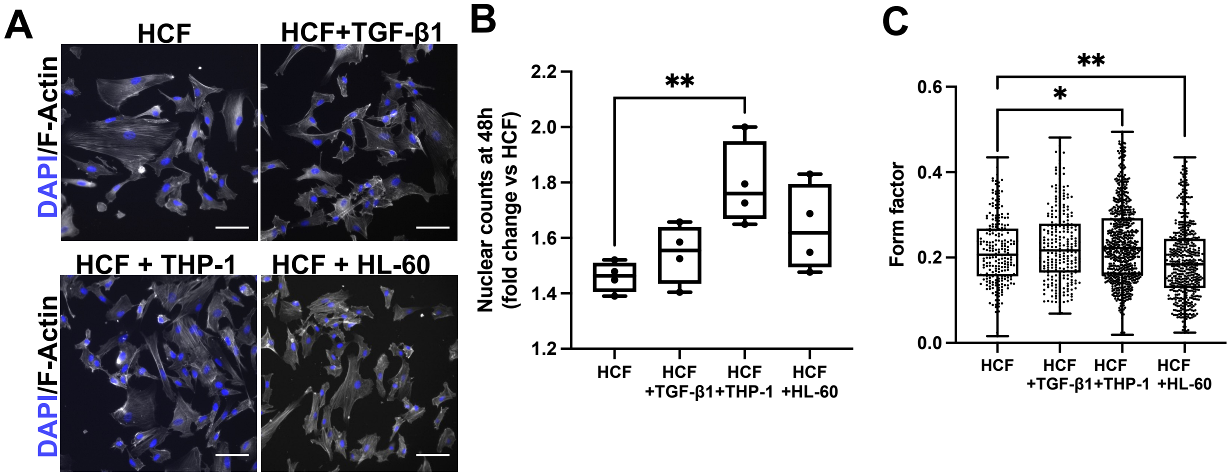

Supplementary Figure 2: HCF co-culture with THP-1 cells in 2D culture leads to increased HCF proliferation and elongation.
(A) Representative images of nuclei (blue; DAPI) and F-actin (white, Alexa-488) staining in HCF alone, treated with TGF-β1 or co-cultured immune cells for 24h. Scale bars represent 50μm. Quantification of fold change of **(B)** cell number at 48h vs. 0h (n=4 independent experiments, each data point represents mean from 3 technical replicates per condition per experiment) and **(C)** form factor (morphology, 0 represents fragmented and 1 represents perfect circle. Data from 1 of 3 independent experiments shown; each data point represents a cell) at 24 hours in all conditions. Statistical analysis performed by one-way ANOVA, followed by Dunnett's multiple comparisons test. *p<0.05, **p<0.01.

Supplementary Figure 3

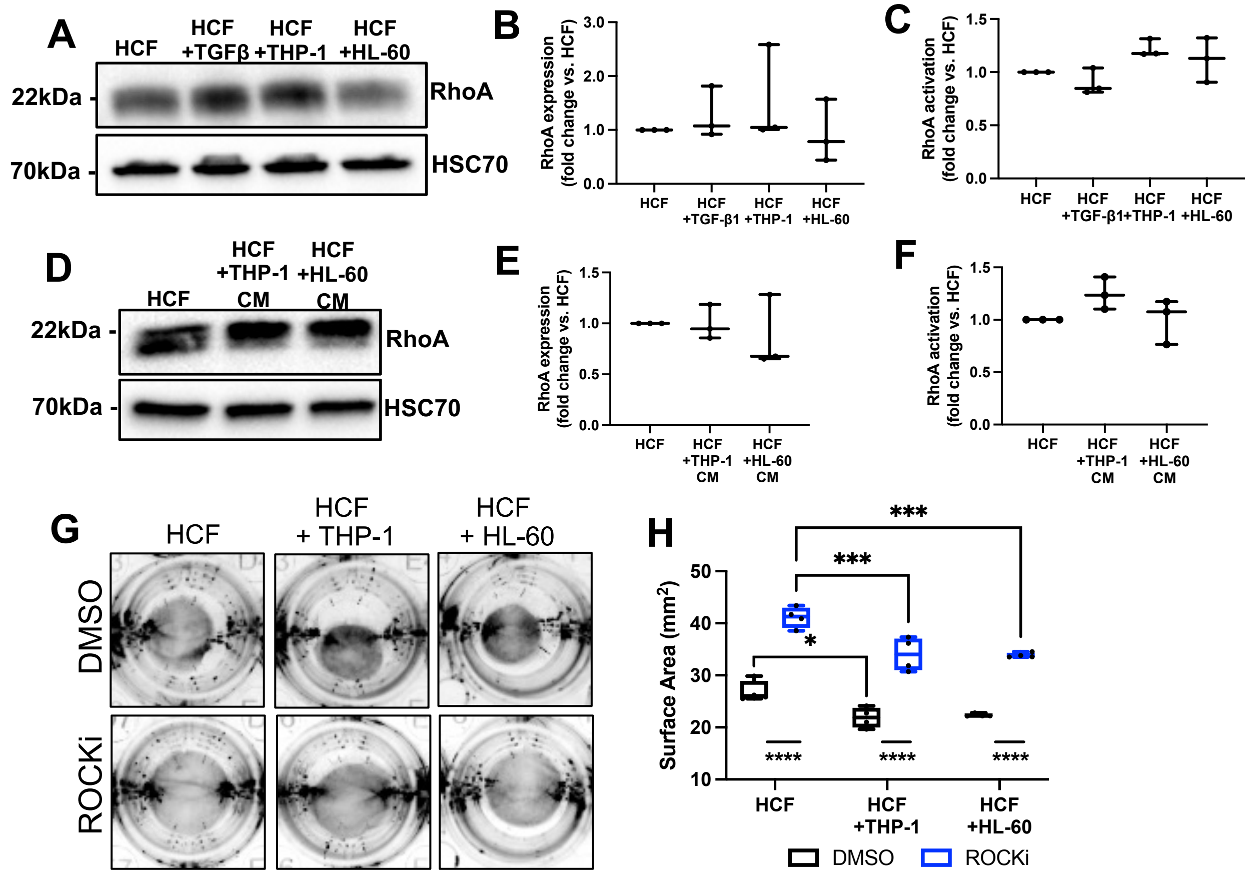

Supplementary Figure 3: HCF RhoA activation is unchanged by co-culture with THP-1 or HL-60 cells

(A) Representative western blots of total RhoA expression in lysates from HCF treated with TGFβ-1 or co-cultured with immune cells and **(B)** quantification of 3 independent experiments from data as in (A). **(C)** RhoA activation G-LISA quantification in HCF treated with TGFβ-1 or co-cultured with immune cells. Means from 3 independent experiments shown. **(D)** Representative western blots of total RhoA expression in lysates from HCF treated with immune cell conditioned media (CM) and **(E)** quantification of total RhoA western blots from 3 independent experiments as in (D). **(F)** RhoA activation G-LISA quantification in HCF treated with immune cell CM. Means from 3 independent experiments shown. **(G)** Representative images of collagen gels with HCF co-cultured with immune cells, with or without ROCK inhibitor treatment at day 3 and quantification of collagen gel surface area in **(H).** Data shown is from one experiment (4 samples per condition), representative of 3 independent experiments; statistical analysis performed by (B,C,E,F) one-way ANOVA with Dunnett's multiple comparisons test (H) two-way ANOVA, followed by Bonferroni's multiple comparisons test. **$p<0.01$, ***$p<0.001$, ****$p<0.0001$.

Supplementary Figure 4

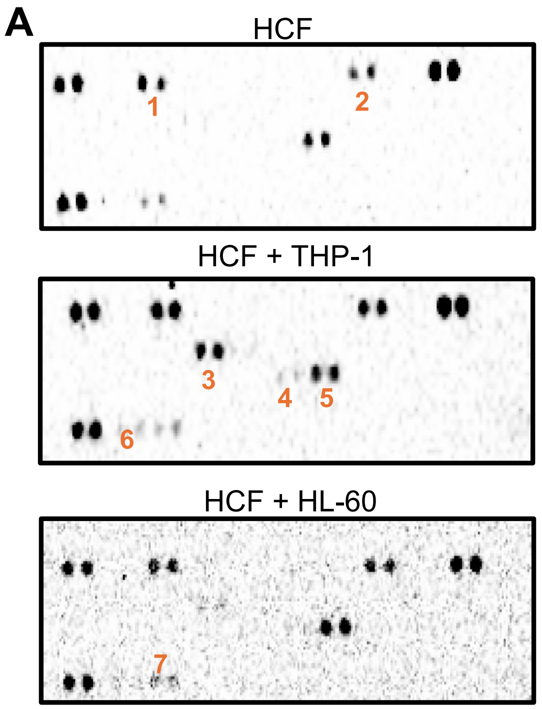

1	CCL2/MCP1
2	CXCL1/GROα
3	G-CSF
4	IL-6
5	IL-8
6	MIF
7	Serpin E1/PAI-1

Supplementary Figure 4: Cytokine abundance in HCF conditioned media **(A)** Representative images of cytokine array membranes from indicated conditions**. (B)** table of cytokines detected, each pair of dots represent the same cytokine; numbers refer to annotated spots in (A).

Supplementary Figure 5

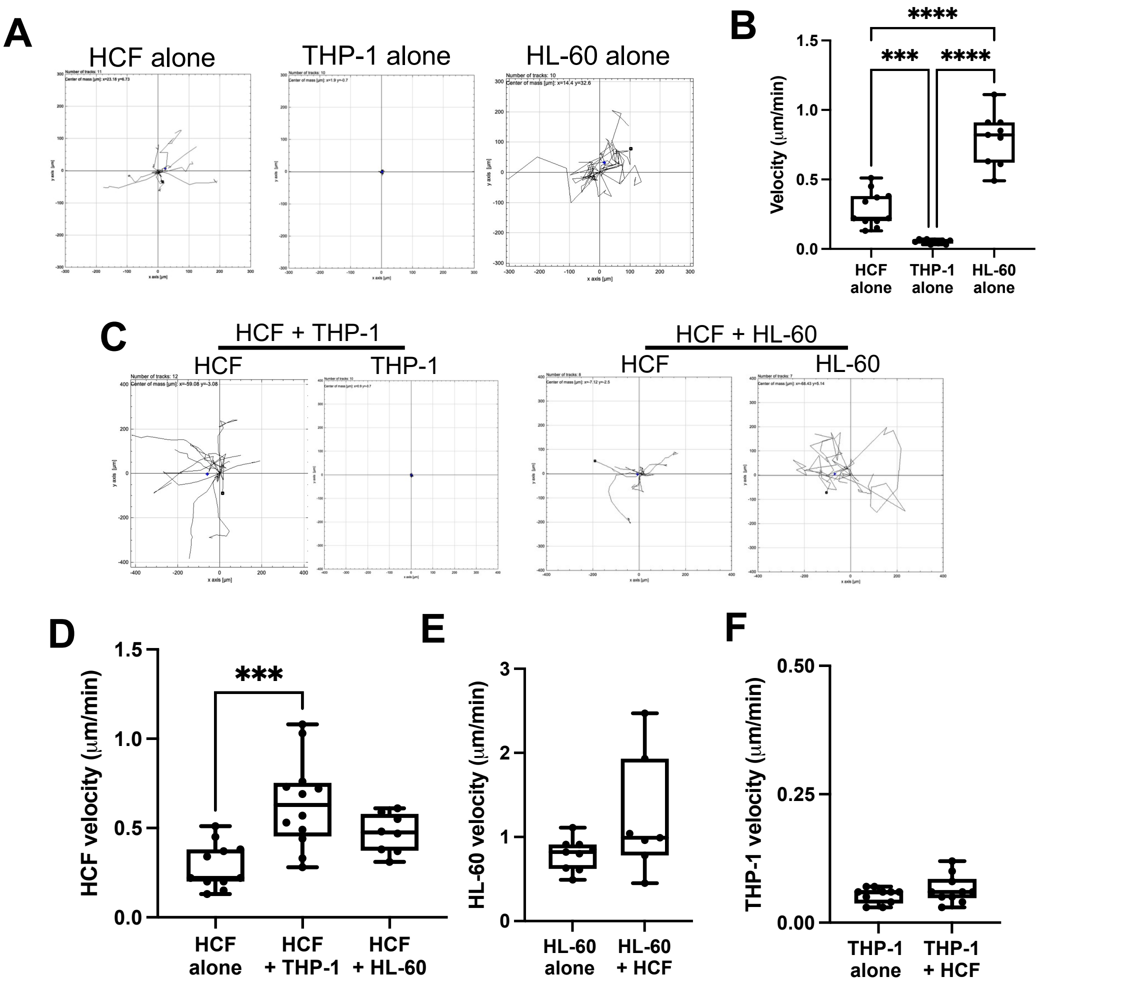

Supplementary Figure 5: HL-60 cells promote HCF migration in 3D collagen gels. (A) Representative migration plots of HCFs, THP-1 and HL-60 cells in collagen gels and **(B)** quantification of velocity of each cell type over imaging period **(C)** Representative migration plots of HCF, THP-1 and HL-60 cells in collagen gels in specified co-cultures. Quantification of velocity over imaging period of **(D)** HCF with and without immune cells, **(E)** HL-60 without and without HCF and **(F)** THP-1 with and without HCF. Data shown is one experiment (n=7-12 movies per condition; each data point in graphs represents mean velocity from all cells within a field of view), representative of three independent experiments; statistical analysis performed by one-way ANOVA, followed by Tukey's multiple comparisons test (E, F) or unpaired T-test (G, H). ***p<0.001, ****p<0.0001.
